# Breast cancer risk and drinking water contaminated by wastewater: a case control study

**DOI:** 10.1186/1476-069X-5-28

**Published:** 2006-10-06

**Authors:** Julia Green Brody, Ann Aschengrau, Wendy McKelvey, Christopher H Swartz, Theresa Kennedy, Ruthann A Rudel

**Affiliations:** 1 Silent Spring Institute, 29 Crafts Street, Newton, MA 02458, USA; 2 Department of Epidemiology, Boston University School of Public Health, 715 Albany Street, Talbot Building, Boston, MA 02118, USA

## Abstract

**Background:**

Drinking water contaminated by wastewater is a potential source of exposure to mammary carcinogens and endocrine disrupting compounds from commercial products and excreted natural and pharmaceutical hormones. These contaminants are hypothesized to increase breast cancer risk. Cape Cod, Massachusetts, has a history of wastewater contamination in many, but not all, of its public water supplies; and the region has a history of higher breast cancer incidence that is unexplained by the population's age, in-migration, mammography use, or established breast cancer risk factors. We conducted a case-control study to investigate whether exposure to drinking water contaminated by wastewater increases the risk of breast cancer.

**Methods:**

Participants were 824 Cape Cod women diagnosed with breast cancer in 1988–1995 and 745 controls who lived in homes served by public drinking water supplies and never lived in a home served by a Cape Cod private well. We assessed each woman's exposure yearly since 1972 at each of her Cape Cod addresses, using nitrate nitrogen (nitrate-N) levels measured in public wells and pumping volumes for the wells. Nitrate-N is an established wastewater indicator in the region. As an alternative drinking water quality indicator, we calculated the fraction of recharge zones in residential, commercial, and pesticide land use areas.

**Results:**

After controlling for established breast cancer risk factors, mammography, and length of residence on Cape Cod, results showed no consistent association between breast cancer and average annual nitrate-N (OR = 1.8; 95% CI 0.6 – 5.0 for ≥ 1.2 vs. < .3 mg/L), the sum of annual nitrate-N concentrations (OR = 0.9; 95% CI 0.6 – 1.5 for ≥ 10 vs. 1 to < 10 mg/L), or the number of years exposed to nitrate-N over 1 mg/L (OR = 0.9; 95% CI 0.5 – 1.5 for ≥ 8 vs. 0 years). Variation in exposure levels was limited, with 99% of women receiving some of their water from supplies with nitrate-N levels in excess of background. The total fraction of residential, commercial, and pesticide use land in recharge zones of public supply wells was associated with a small statistically unstable higher breast cancer incidence (OR = 1.4; 95% CI 0.8–2.4 for highest compared with lowest land use), but risk did not increase for increasing land use fractions.

**Conclusion:**

Results did not provide evidence of an association between breast cancer and drinking water contaminated by wastewater. The computer mapping methods used in this study to link routine measurements required by the Safe Drinking Water Act with interview data can enhance individual-level epidemiologic studies of multiple health outcomes, including diseases with substantial latency.

## Background

Unexplained geographic variation in breast cancer incidence suggests the possibility of one or more regional environmental risk factors. Two sets of common pollutants – animal mammary carcinogens and endocrine disrupting compounds (EDCs) – have been identified as biologically plausible candidates [1-3]. Many EDCs – including compounds in banned and current use pesticides; phenolic compounds in some detergents and plastics; and parabens in some cosmetics and prepared foods – have been shown in animal and cell studies to mimic estrogen, which is an established breast cancer risk factor [1,2,4]. Mammary carcinogens include benzene and other organic solvents; polycyclic aromatic hydrocarbons (PAHs), which are products of combustion; some pesticides; and some pharmaceuticals and endogenous hormones (e.g., estrogen) [3,5-7].

Because EDCs and mammary carcinogens are in common use, they are found in wastewater and run-off from developed land. Excreted natural hormones and pharmaceuticals further add to the estrogenic burden in wastewater. Thus, drinking water from sources impacted by wastewater and run-off is a potential pathway of human exposure [8-11] that may vary regionally by water supply.

Cape Cod, Massachusetts, a peninsula of sandy glacial deposits extending into the Atlantic Ocean, has a history of wastewater and land use impact in many, but not all, of its 18 public water supplies, which draw water from more than 100 shallow wells in the aquifer that underlies the region [12-14]. Public supplies serve 82% of housing units [15] for the year-round population of 222,000 residents and more than 500,000 in summer [16,17]. Extensive residential and commercial development since the 1980s has replaced forests and wetlands on land that recharges the aquifer; and 85% of residences rely on septic systems for wastewater disposal [18]. Sandy soils allow septic system effluent and run-off of surface pollutants to leach into groundwater. A US Geological Survey (USGS) modeling study of 15 public supply wells on the Cape showed that up to 26% of the water being pumped originated as septic system discharges [19].

In previous research, we demonstrated that septic wastes are a source of exposure to EDCs. We reported estrogenic detergent degradation products (alkylphenols) in Cape Cod septage at the highest levels that have been reported in wastewater and substantial concentrations in groundwater impacted by wastewater [11]. We found low levels of these compounds in 6 of 28 private drinking water wells, providing evidence of a pathway of exposure to EDCs from drinking water in this region. In an earlier sampling effort that had higher detection limits and fewer target compounds, we did not find alkylphenols in five samples from public drinking water wells [20].

Cape Cod also has a history of unexplained elevated breast cancer incidence, documented in three different data sets. In the Cape Cod Breast Cancer and Environment Study, women with longer years of residence on Cape Cod had higher breast cancer risk, after taking into account established breast cancer risk factors and education, a marker of socioeconomic status [21]. In a cross-sectional analysis of Collaborative Breast Cancer Study data, Cape Cod women aged 50 – 74 had 21% higher breast cancer risk than other Massachusetts women, controlling for established and suggested risk factors [20]; and Massachusetts Cancer Registry (MCR) surveillance data show age-adjusted breast cancer incidence on Cape Cod was about 20% higher than the rest of the state for 1982–1994 [22], while mammography use was similar or lower [23]. Mortality was not elevated during those years [22].

A possible association between breast cancer risk and EDCs and mammary carcinogens in drinking water cannot be assessed directly, because most EDCs and mammary carcinogens have not been routinely measured in drinking water over the likely etiologic period for diagnosed breast cancers. However, previous research supports the use of nitrate nitrogen (nitrate-N) measurements as a proxy for wastewater impacts [24,25]. Nitrate-N, an oxidation product of nitrogen in wastewater, can be a useful indicator of wastewater impact to groundwater, because it typically travels at about the rate of groundwater through the aquifer. It has been measured regularly in public drinking water since the early 1970s to meet federal requirements. Although in other regions, agricultural wastes and fertilizer are sources of nitrate-N, domestic wastewater is the dominant source in residential areas on Cape Cod [26,27].

Based on the biologically plausible hypothesis that EDCs and mammary carcinogens increase breast cancer risk, the presence of these contaminants in wastewater and surface run-off, the history of wastewater impacts on Cape Cod public drinking water supplies, and the concerns of community residents, we investigated the association between breast cancer risk and contaminated public drinking water in a case-control study of Cape Cod women diagnosed with breast cancer, using historical nitrate-N levels as an indicator of contaminant levels.

## Methods

### Study population

We conducted a case-control study of invasive breast cancer diagnosed in 1988–1995 among permanent residents of Cape Cod. The selection and enrollment of the study population have been described previously [21,28]. Briefly, women who were permanent residents of Cape Cod for at least six months at the time of an invasive breast cancer diagnosis in 1988–1995 and whose diagnosis was reported to the MCR were eligible cases. Controls were selected from women who were permanent residents of Cape Cod for at least six months in 1988 – 1995. They were frequency matched to cases on date of birth in decades and vital status. Living controls under age 65 were selected using random digit dialing. Living controls aged 65 and older were selected randomly from lists of Medicare beneficiaries from the Centers for Medicare and Medicaid Services (CMS). Deceased controls were selected randomly from Massachusetts Department of Vital Statistics death certificates for appropriately aged residents who died after January 1988 and were frequency matched to cases by age and year of death. Next-of-kin were identified from death certificates and obituaries as proxies for deceased women. Cases diagnosed in 1988–1993 in eight towns and their controls were interviewed in 1997–1998 in a study of breast cancer and perchloroethylene (PCE) in drinking water [29]. Cases diagnosed in 1994–1995 in those towns and in 1988–1995 in the remaining seven Cape Cod towns and their controls were interviewed in 1999–2000.

The study protocol and permissions to use confidential data were approved by the Massachusetts Department of Public Health Human Research Review Committee, MCR, CMS, National Death Index, the Boston University Institutional Review Board, and review boards at hospitals where cases were diagnosed. Informed consent was obtained at the outset of telephone interviews.

Among 1578 eligible cases, 1165 (74%) participated, 228 (14%) were never located or contacted, and 185 (12%) refused to participate. Among 1503 eligible controls, 1016 (68%) participated. After interviewing, controls were assigned reference years according to the distribution of diagnosis years among the cases. If a control had moved to Cape Cod after the randomly assigned reference year, she was considered ineligible and excluded from analyses (n = 71). Exposures after diagnosis (for cases) or reference year (for controls) were excluded as not etiologically relevant.

Study participants were selected for the drinking water investigation if all of the residences where they lived on Cape Cod during the 16 years prior to diagnosis or index year were geocodable and served by a public drinking water supply. We excluded 296 cases and 225 controls who lived in homes served by a private well during some or all of the 16 years prior to diagnosis or index year. We limited the analysis to women served by public water supplies, because routine monitoring of public wells provides a systematic source of exposure data. We limited the exposure period to the 16 years before diagnosis for reasons, described below, related to availability of water data. Although we excluded women in Cape Cod residences with private wells, because unmeasured exposure may occur in these residences, we included women who were off Cape Cod during a part of the exposure period, because wastewater contamination of drinking water is unlikely in the areas from which most of the women migrated. Among eligible women, 6 cases and 21 controls were excluded because they had non-geocodable addresses; and 22 cases and 6 controls were excluded because they were missing information on education or age at first birth, leaving 824 cases and 745 controls for analysis.

### Interviews

We interviewed women by telephone about their home drinking water source and water use habits, their home addresses on Cape Cod and dates of residence, and established and hypothesized breast cancer risk factors. Interviews included family history of breast cancer (in a mother, sister, daughter), menstrual and reproductive history, height and weight, alcohol and tobacco use, physical activity, pharmaceutical hormone use, and education. Interviews also included water use behaviors at each Cape Cod residence, including use of bottled drinking water and of carbon or charcoal water filters.

### Geocoding of residential addresses

Women's residential addresses on Cape Cod were geocoded to a latitude and longitude. We mapped addresses wherever possible to the center of a visible residential rooftop using town parcel maps and the state base map for Massachusetts, which is 0.5 m resolution Color Ortho Imagery [30]. Mapping of geocoded points to a visible residential rooftop was done using ESRI's ArcGIS ArcINFO version 8.3 and ArcView 3.1 [31]. If the house could not be identified on the orthophotos, the address was geocoded, if possible, to the center of a cluster of rooftops in the parcel or the center of the parcel if no rooftops were visible. Addresses identified by a cross street or landmark rather than a numerical address were geocoded using the following rules in descending order: to the nearest rooftop, to the center of the nearest parcel, or to the middle of the street if the street was less than a mile long. Addresses that could not be located with this level of accuracy were excluded from analyses. "Move in" and "move out" years were linked to each address to allow for analysis of changing exposure levels during intervening years. Additional detail on this method is found in Brody et al. [28]. We did not collect information about street addresses off Cape Cod.

### Drinking water exposure assessment

We assessed wastewater impacts on public drinking water using measured levels of nitrate-N in supply wells as a proxy. We used the Cape Cod Study GIS to link the nitrate-N measurements with information about the drinking water distribution systems and individual interview data. As an alternative water quality indicator in addition to nitrate-N, we investigated land use in the public water supply zones of contribution (ZOCs, the surface areas that contribute recharge to the wells). Additional details of data sources, exposure assessment methods, and Cape Cod historical drinking water quality are found in Swartz et al. [14].

Cape Cod is served by 18 public water districts comprising 132 wells and one surface supply that were operating during the study's exposure period. (For simplicity, we use "wells" to refer to the supply sources even though there is one surface supply.) The average population size served by these eighteen utilities was 8,594 people, with a range from 1,320 to 22,799, based on the 1990 US census.

Routine nitrate-N measurement in public water supplies began in 1972 as required by the Safe Drinking Water Act; so we assessed exposures from 1972 to 1995, the most recent diagnosis year in the study. Annual nitrate-N concentrations were obtained from Cape Cod Commission, Massachusetts Department of Environmental Protection (MA DEP), and individual water districts. The surface supply was treated in the same way as a well in the analysis. Missing values were interpolated by linear regression of existing data points. If the earliest nitrate-N measurement was later than 1972, we extrapolated back one year, because wells were typically in operation during the year prior to the first nitrate-N measurement. On average, 19% of a district's nitrate-N measurements were interpolated or extrapolated, usually over a one-year gap. Previous analysis showed that nitrate-N values were rising linearly during the period 1972–1995, supporting this method [14]. Because our goal was to assess nitrate-N from human activities, we calculated anthropogenic nitrate-N as the annual concentration (mg/L nitrate nitrogen) after subtracting 0.2 mg/L, which is the maximum detected in un-impacted wells in the region [24,25]. Resulting scores will be referred to as "excess nitrate-N." Wells with less than 0.2 mg/L were scored zero. Next we calculated annual excess nitrate-N for each water supply district by weighting the scores for individual wells by the percentage contribution of that well to the district's total pumping volume based on pumping data from USGS [32,33], MA DEP [34], and individual districts.

We assigned residential addresses to the appropriate water district using maps of distribution pipes obtained from each district and digitized using ESRI's ArcINFO version 7 [35]. For residences reported in interviews to be on public water, we assigned the residence to the district served by the nearest distribution pipe. If interview data about water source was missing, we assigned the residence to a water district if it was within 75 m of a distribution pipe (Cambareri, personal communication 1997).

Because nitrate-N measurement began in 1972, sixteen years of exposure data are available for women with the earliest diagnosis/index year (1988) and 23 years for women with the most recent diagnosis/index year (1995). To avoid assigning higher exposures to women simply because more years of data were available, we limited exposure assessment to the 16 years prior to diagnosis or index year. Within this 16-year period, we calculated (1) average annual excess nitrate-N levels at each woman's Cape Cod residences, (2) the sum of excess nitrate-N concentrations in each of the 16 years, and (3) the number of years with excess nitrate-N levels greater than 1 mg/L.

Tap water quality is likely to vary spatially at residences within some districts, depending on the heterogeneity of source water quality at the wells, the configuration of the distribution system, and other factors. Water districts were unable to provide models of intra-district variation. However, for the district-level exposure measures we used, we expect exposure misclassification to be lower in districts where the concentration of wastewater contaminants at the tap is likely to be homogeneous; because (1) water from all the wells is redistributed from a single point, such as a water tower, or a small number of sources, and it is expected to be homogeneously mixed, (2) water quality is similar in all the supply wells, or (3) there are no spatially distinct areas of "high" or "low" contaminant wells. We limited some analyses to districts that met these criteria. Districts with a single distribution point were Barnstable Fire District, North Sagamore, and South Sagamore; and Yarmouth also reported complete mixing. Districts where all wells showed little or no nitrate-N impact above background were Bourne, Brewster, and Orleans. Other districts (Buzzards Bay, Centerville-Osterville-Marstons Mills, Cotuit, Dennis, Falmouth, Harwich Mashpee, and Provincetown-Truro) had a range of nitrate-N levels, but "high" and "low" wells were in close proximity to each other, supporting an assumption of homogeneous mixing.

We used maps derived from aerial photography in 1971, 1984, and 1990 to assess drinking water quality based on land use in the drinking water well ZOCs. Residential and commercial lands are indicators of septic system impacts on groundwater and drinking water. Cranberry bogs, other agricultural land, rights of way, and golf courses were evaluated as indicators of routine pesticide use. We calculated for each year in the aerial photographs the fraction of the area of the ZOC in commercial or residential use (i.e., with septic systems) or where pesticides would have been routinely applied. We used linear interpolation to calculate land use fractions for each intervening year and extrapolation for later dates. The median residential fraction in Cape ZOCs rose from 2% in 1951 to 11% in 1971 and 23% in 1990, with 80% residential land in the most-impacted ZOC. The median commercial and pesticide use fractions were 4% and about 6% in 1990. The commercial fraction increased from 2% in 1971, while the pesticide use fraction was stable. We used the same procedure as for the nitrate-N measurements to aggregate exposures across wells within a supply and across an individual's years of exposure. We limited the analysis to exposures during the 16 years prior to diagnosis or index year in order to parallel the nitrate-N analysis.

Exposure to contaminants in a public water supply is modified by personal behaviors including the volume of drinking water consumed directly and used in cooking, and inhalation from bathing, showering, dishwashers, and clothes washing. Thus, in some analyses, we considered women who never used bottled water or a water filter more exposed.

## Data analysis

We used unconditional logistic regression to calculate crude and adjusted odds ratios and 95% confidence intervals for exposure to public drinking water contaminants and breast cancer. The following matching variables and potential confounders were controlled in all adjusted odds ratio analyses based on the research design, well-established breast cancer risk factors, and the completeness of data: age as a continuous term, birth decade (6 categories), PCE vs. Cape Cod Study, vital status, year of diagnosis/reference year, prior breast cancer, age at birth of first child (30+ or nulliparous vs. < 30), family history of breast cancer in a first degree female relative, and education (5 categories). Additional potential confounders were also considered: mammography use, medical radiation, lactation, hormone replacement therapy, oral contraceptive use, diethylstilbestrol exposure, body mass index, smoking, alcohol consumption, teen and adult physical activity, race, marital status, and religion. None of these variables changed the OR estimates by ≥ 10%, and they were not included in final models. We considered length of residence on Cape Cod (years) during the exposure period as a possible confounder; because length of residence on Cape Cod is associated with breast cancer [21] and because drinking water exposure calculations are a function of the number of years women lived on Cape Cod within the 16-year drinking water exposure period. Years on Cape Cod squared was also included because it added significantly to the overall fit of the model. Finally, we considered possible confounding by the accidental exposure to PCE from certain water distribution pipes in the region [29] in analyses stratified by this exposure for the 713 study participants for whom it was assessed.

## Results

Women in the overall Cape Cod Breast Cancer and Environment Study were predominantly white and aged 60 to 80 with a high school or higher level of education. Cases were on average 67 (SD = 12) years old and controls 65 (SD = 13). Both cases and controls lived on average approximately 17 years (SD = 12) on Cape Cod. As expected, nulliparity and age 30 years or more at the birth of a first child, and family history of breast cancer in a mother, sister, or daughter were associated with breast cancer risk. Characteristics of study participants whose Cape Cod residences were served by public water supplies during the 16 years prior to diagnosis or index year are shown in Table 1. Among these women, 455 cases (55%) and 400 controls (54%) were interviewed in the Cape Cod Study and the remainder in the PCE Study. At the time of interview, 227 cases (28%) and 185 controls (25%) were deceased. Proxies for these women were predominantly their spouses. During the exposure assessment period (16 years prior to diagnosis or index year), 381 cases (46%) and 310 controls (42%) were always on Cape Cod public drinking water supplies; while 172 cases (21%) and 199 controls (27%) were on Cape Cod public supplies for fewer than seven years.

**Table 1:** Characteristics of cases and controls whose Cape Cod residences were served by public water supplies.

Characteristic	Cases (n = 824) No. (%)	Controls (n = 745) No. (%)	Adjusted OR (95% CI)^a^
Family history of breast cancer^b^			
No	620 (75)	602 (81)	1.0 (reference)
Yes	204 (25)	143 (19)	1.4 (1.1 – 1.7)
Age at birth of first child			
Under 30	510 (62)	530 (71)	1.0 (reference)
Nulliparous, or 30+	314 (38)	215 (29)	1.6 (1.3 – 2.0)
Prior breast cancer diagnosis			
No	761 (92)	680 (91)	1.0 (reference)
Yes	63 (8)	65 (9)	0.8 (0.5 – 1.1)
Highest grade completed			
Less than high school graduate	54 (7)	75 (10)	0.6 (0.4 – 0.9)
High school graduate	280 (34)	237 (32)	1.0 (reference)
Some college/vocational	249 (30)	221 (30)	1.0 (0.7 – 1.2)
College graduate	140 (17)	131 (18)	0.9 (0.7 = 1.3)
Graduate study/degree	101 (12)	81 (11)	1.1 (0.8 – 1.6)

^a ^Adjusted for all other variables in the table and diagnosis/reference year, age at diagnosis/reference year, birth decade, study, and vital status

^b ^Breast cancer diagnosis in a mother, sister, or daughter.

Very few women were unexposed to drinking water impacted by wastewater. Only 8 (1.1%) controls and 11 (1.3%) cases lived in residences that never received water from Cape supplies with nitrate-N in excess of background. About 32% of cases and controls were exposed to excess nitrate-N of more than 1 mg/L in at least one year, and 9% were exposed at this level for six or more years. About 9% of cases and 10% of controls had average annual excess nitrate-N above 1 mg/L.

Odds ratio analyses showed no pattern of association between water district nitrate-N measurements and breast cancer (Table 2). Crude and adjusted ORs were similar, so only adjusted ORs are shown. Results were similar when analysis was limited to the 1305 women in water districts with homogenous water quality within the district (Table 3). Risk was slightly elevated only for the highest exposure group for average annual excess nitrate-N, and the elevation was not statistically significant (adjusted OR = 1.2; 95% CI 0.5–3.1). No association was seen when the analysis was limited to women who were on Cape Cod public supplies during the full 16-year period for which we assessed exposures or for shorter periods (< 7, 7 to < 16 years), and we found no association in analyses of exposure 0 to < 6, 6 to < 11, or 11 to 16 years prior to index year (not shown). Results were similar when limited to non-proxy respondents and were unchanged by combining exposures into three rather than five exposure groups (not shown). Average annual nitrate-N levels were significantly lower among women who had lived in homes accidentally exposed to PCE from water distribution pipes (*p *< .0001), but odds ratios for nitrate-N exposure were not appreciably changed by including PCE exposure in the model (not shown). Sixty percent of cases and 61% of controls reported that they never regularly used bottled water or a carbon/charcoal filter at a Cape Cod residence. Odds ratios for district nitrate-N exposure and breast cancer in this group were similar to the overall study population (not shown).

**Table 2:** Associations between wastewater impacted public drinking water and breast cancer.

	Cases	Controls	Adjusted OR (95% CI)^a^	Adjusted OR (95% CI)^b^
Average annual excess nitrate-N concentration (mg/L)				
0 to < .3	327	287	1.0 (reference)	1.0 (reference)
.3 to < .6	201	175	1.0 (0.7 – 1.3)	0.9 (0.7 – 1.3)
.6 to < .9	155	152	0.9 (0.6 – 1.2)	0.8 (0.6 – 1.1)
.9 to < 1.2	120	112	0.9 (0.6 – 1.2)	0.9 (0.6 – 1.2)
≥ 1.2	21	19	0.9 (0.5 – 1.7)	1.0 (0.5 – 1.9)
Sum of annual excess nitrate-N concentrations (mg/L)				
0 to < .01	19	18	0.8 (0.4 – 1.6)	0.9 (0.5 – 1.9)
.01 to < .1	45	42	0.9 (0.6 – 1.5)	1.1 (0.7 – 1.7)
.1 to < 1	119	118	0.9 (0.7 – 1.2)	1.1 (0.8 – 1.5)
1 to < 10	474	418	1.0 (reference)	1.0 (reference)
≥ 10	167	149	1.0 (0.8 – 1.3)	0.9 (0.7 – 1.2)	
Number of years exposed to excess nitrate-N > 1 mg/L				
0	562	504	1.0 (reference)	1.0 (reference)
< 2	58	52	1.0 (0.7 – 1.5)	1.1 (0.7 – 1.6)
2 to < 4	93	77	1.0 (0.7 – 1.4)	1.0 (0.7 – 1.4)
4 to < 6	36	44	0.8 (0.5 – 1.2)	0.7 (0.5 – 1.2)
6 to < 8	50	43	1.0 (0.6 – 1.5)	0.9 (0.6 – 1.4)
≥ 8	25	25	0.9 (0.5 – 1.7)	0.9 (0.5 – 1.5)
OR odds ratio. CI confidence interval.				

^a ^Adjusted for diagnosis/reference year, age at diagnosis/reference year, birth decade, study, vital status, previous breast cancer diagnosis, age at first birth, family history of breast cancer, and education.

^b ^Adjusted for variables in analysis (a), years on Cape Cod and years on the Cape squared.

**Table 3:** Associations between wastewater impacted public drinking water in homogenous supplies and breast cancer.

	Cases	Controls	Adjusted OR (95% CI)^a^	Adjusted OR (95% CI)^b^
Average annual excess nitrate-N concentration (mg/L)				
0 to < .3	280	250	1.0 (referent)	1.0 (referent)
.3 to < .6	163	141	1.0 (0.7 – 1.3)	1.0 (0.7 – 1.3)
.6 to < .9	149	144	0.9 (0.6 – 1.2)	0.9 (0.6 – 1.2)
.9 to < 1.2	78	79	0.8 (0.5 – 1.2)	0.8 (0.5 – 1.2)
≥ 1.2	12	9	1.2 (0.5 – 2.9)	1.2 (0.5 – 3.1)
Sum of annual excess nitrate-N concentrations (mg/L)				
0 to < .01	18	17	0.8 (0.4 – 1.6)	0.9 (0.4 – 1.9)
.01 to < .1	43	41	0.9 (0.5 – 1.4)	1.0 (0.6 – 1.7)
.1 to < 1	104	104	0.9 (0.6 – 1.2)	1.1 (0.8 – 1.5)
1 to < 10	390	344	1.0 (referent)	1.0 (referent)
≥ 10	127	117	1.0 (0.7 – 1.3)	0.9 (0.6 – 1.2)
Number of years exposed to excess nitrate-N > 1 mg/L				
0	479	433	1.0 (referent)	1.0 (referent)
< 2	50	47	0.9 (0.6 – 1.4)	1.0 (0.6 – 1.5)
2 to < 4	87	69	1.1 (0.7 – 1.6)	1.0 (0.7 – 1.5)
4 to < 6	66	74	0.8 (0.5 – 1.2)	0.7 (0.5 – 1.1)

^a ^Adjusted for diagnosis/reference year, age at diagnosis/reference year, birth decade, study, vital status, previous breast cancer diagnosis, age at first birth, family history of breast cancer, and education.

^b ^Adjusted for variables in analysis [a] and years on Cape Cod and years on the Cape squared.

We found no consistent pattern of associations between breast cancer and the fraction of land use in the supply well ZOCs that was residential, commercial, or in pesticide use areas (Table 4). For total land use (the sum of residential, commercial, and pesticide fractions), odds ratios were slightly elevated for nearly all categories compared with the lowest land use fraction (adjusted OR = 1.4; 95% CI 0.8–2.4 for highest compared with lowest). Odds ratios were not statistically significant (*p *< 0.05) and did not increase for increasing land use fractions. Results were similar in analyses limited to women on homogeneous water supplies (Table 5).

**Table 4:** Associations between land use in recharge areas for drinking water wells and breast cancer.

Fraction of Recharge Area in Specified Land Use	Cases	Controls	Adjusted OR (95% CI)^a^	Adjusted OR (95% CI)^b^
Residential				
0 to < .5	252	262	1.0 (reference)	1.0 (reference)
.5 to < 1	243	195	1.3 (1.0–1.7)	1.1 (0.8–1.5)
1 to < 1.5	157	136	1.2 (0.9–1.6)	0.9 (0.7–1.3)
1.5 to < 2	125	106	1.3 (0.9–1.7)	1.0 (0.7–1.4)
≥ 2	47	46	1.1 (0.7–1.7)	0.8 (0.5–1.4)
Commercial				
0 to < .5	671	611	1.0 (reference)	1.0 (reference)
.5 to < 1	58	61	0.9 (0.6–1.4)	0.8 (0.6–1.3)
1 to < 1.5	17	16	1.1 (0.5–2.2)	1.2 (0.6–2.4)
1.5 to < 2	19	11	1.7 (0.8–3.7)	1.7 (0.8–3.6)
2 to < 2.5	10	5	2.1 (0.7–6.4)	2.0 (0.7–6.2)
2.5 to < 3	25	22	1.2 (0.7–2.2)	1.1 (0.6–2.0)
≥ 3	24	19	1.2 (0.6–2.2)	1.0 (0.5–1.9)
Pesticide application area				
0 to < .5	303	309	1.0 (reference)	1.0 (reference)
.5 to < 1	240	187	1.3 (1.0–1.7)	1.1 (0.8–1.5)
1 to < 1.5	238	211	1.2 (0.9–1.6)	0.9 (0.7–1.3)
≥ 1.5	43	38	1.2 (0.8–2.0)	1.0 (0.6–1.6)
Total^c^				
0 to < .5	86	112	1.0 (reference)	1.0 (reference)
.5 to < 1	120	101	1.6 (1.1–2.4)	1.4 (0.9–2.2)
1 to < 1.5	75	72	1.4 (0.9–2.2)	1.2 (0.8–2.0)
1.5 to < 2	95	84	1.6 (1.0–2.4)	1.3 (0.8–2.2)
2 to < 2.5	196	155	1.8 (1.2–2.6)	1.4 (0.8–2.4)
2.5 to < 3	51	48	1.4 (0.9–2.4)	1.1 (0.6–2.1)
3 to < 3.5	109	97	1.6 (1.1–2.4)	1.2 (0.7–2.2)
≥ 3.5	92	76	1.7 (1.1–2.7)	1.4 (0.8–2.4)

^a ^Adjusted for diagnosis/reference year, age at diagnosis/reference year, birth decade, study, vital status, previous breast cancer diagnosis, age at first birth, family history of breast cancer, and education.

^b ^Adjusted for variables in analysis [a] and years on Cape Cod and years on the Cape squared.

^c ^Residential plus pesticide use area plus commercial.

**Table 5:** Associations between land use in recharge areas for homogenous drinking water supplies and breast cancer.

Fraction of Recharge Area in Specified Land Use	Cases	Controls	Adjusted OR (95% CI)^a^	Adjusted OR (95% CI)^b^
Residential				
0 to < .5	201	211	1.0 (reference)	1.0 (reference)
.5 to < 1	234	184	1.4 (1.0–1.9)	1.1 (0.8–1.6)
1 to < 1.5	139	122	1.2 (0.8–1.6)	0.9 (0.6–1.3)
1.5 to < 2	99	90	1.2 (0.8–1.7)	0.9 (0.6–1.4)
≥ 2	9	16	0.7 (0.3–1.6)	0.5 (0.2–1.2)
Commercial				
0 to < .5	587	533	1.0 (reference)	1.0 (reference)
.5 to < 1	49	54	0.9 (0.6–1.4)	0.8 (0.5–1.3)
1 to < 1.5	12	8	1.6 (0.6–4.1)	1.7 (0.7–4.4)
1.5 to < 2	10	7	1.4 (0.5–3.9)	1.4 (0.5–3.7)
2 to < 2.5	7	3	2.6 (0.6–10.3)	2.3 (0.6–9.3)
2.5 to < 3	17	18	1.0 (0.5–2.0)	0.9 (0.4–1.8)
Pesticide application area				
0 to < .5	234	243	1.0 (reference)	1.0 (reference)
.5 to < 1	178	140	1.3 (1.0–1.8)	1.2 (0.8–1.6)
1 to < 1.5	229	203	1.2 (0.9–1.6)	0.9 (0.7–1.4)
≥ 1.5	41	37	1.2 (0.7–2.0)	0.9 (0.5–1.6)
Total^c^				
0 to < .5	70	93	1.0 (reference)	1.0 (reference)
.5 to < 1	87	72	1.7 (1.1–2.6)	1.5 (0.9–2.4)
1 to < 1.5	69	64	1.5 (0.9–2.4)	1.3 (0.7–2.2)
1.5 to < 2	90	81	1.6 (1.0–2.6)	1.3 (0.7–2.3)
2 to < 2.5	187	149	1.9 (1.3–2.8)	1.4 (0.8–2.6)
2.5 to < 3	46	40	1.5 (0.9–2.6)	1.2 (0.6–2.3)
3 to < 3.5	98	87	1.7 (1.1–2.6)	1.2 (0.6–2.4)
≥ 3.5	35	37	1.4 (0.8–2.5)	1.0 (0.5–2.2)

^a ^Adjusted for diagnosis/reference year, age at diagnosis/reference year, birth decade, study, vital status, previous breast cancer diagnosis, age at first birth, family history of breast cancer, and education.

^b ^Adjusted for variables in analysis [a] and years on Cape Cod and years on the Cape squared.

^c ^Residential plus pesticide use area plus commercial.

## Discussion

Although studies have documented EDC contamination of drinking water [8,11,36], we are not aware of any previous epidemiologic investigations of whether these exposures are associated with breast cancer risk. Results of this population-based case-control study did not show an association between breast cancer diagnosed on Cape Cod in 1988–1995 and residential exposure during the 16 years before diagnosis to drinking water contaminated by anthropogenic nitrate-N, an indicator of wastewater impact. While there was some suggestion of a weak association between total land use in recharge zones and breast cancer risk, the odds ratios were not statistically significant, did not increase with increasing land use fraction, were not elevated for residential or pesticide land use, and were not consistently elevated for commercial land use. Although previous research provides evidence of a number of breast cancer risk factors (pregnancy, hormone replacement therapy, oral contraceptives, and alcohol consumption) with effects during the five years before diagnosis and we hypothesized that EDCs might act as tumor promoters, our results do not support an effect of wastewater-contaminated drinking water during the five years before diagnosis.

Our confidence in the lack of association we observed is tempered by the limitations of our exposure assessment. The range of exposures was limited, with few women unexposed and none exposed at high levels; so we can say nothing about effects of higher exposure levels. This caution is notable, because the odds ratio, though statistically unstable, was elevated for the highest average annual nitrate-N levels in homogeneous supplies and for total land use. Variation in exposures is also limited by the relatively small number of water districts – 18 – included in the study. In addition, assessment was limited to the 16 years before diagnosis and to years when study participants lived on Cape Cod, so we cannot draw conclusions about the effects of earlier exposures or exposures of longer duration. Although we are missing information about off-Cape exposures, we do not think this is a major limitation, because off-Cape exposures to wastewater in drinking water are likely to be low for women who later migrated to the Cape; and off-Cape exposure to other contaminants, such as disinfection byproducts, are unlikely to be correlated with women's Cape Cod exposure.

Though we evaluated the effects of variation in water quality within a district by limiting some analyses to supplies where water at the tap is likely to be similar at geographic locations throughout the district, the measurement of water quality at the well and district level rather than at the tap and at a small number of times per year is a limitation. Using the terminology proposed by Kunzli and Trage [37], this study design would be characterized as "semi-individual." The health outcome and potential confounders were measured at the individual level, and the exposure measure is composed of an individual assessment (the participant's address in each year) and an ecologic component (district-level water quality measurements) assigned to the addresses.

In addition, it is difficult to retrospectively collect information about individual behaviors that affect residential water exposure. Although we found that results were unchanged when we limited the analysis to women who were more exposed to tap water, because they never regularly used bottled water or a filter, this is a crude strategy for incorporating individual behaviors that may modify exposure. Lack of information about exposures off Cape Cod, intra-district variation in water contamination, interpolation of some nitrate-N scores for individual wells, and varying water use behavior are likely to result in non-differential misclassification, which tends to bias odds ratios toward the null where misclassification is not extreme [38].

Finally, our hypothesis that nitrate-N would be associated with breast cancer risk was based on the assumption that because nitrate-N is an established indicator of wastewater impact, it would be a reasonable proxy for impacts from EDCs in wastewater. Our ongoing groundwater research has recently documented that excreted natural estrogens and EDCs do leach from septic systems into groundwater, and they travel and are persistent in the Cape Cod aquifer[39]. Results showed that ammonia-N is correlated with the presence of EDCs at the site we studied. Ammonia-N, another form of inorganic nitrogen found in wastewater plumes where dissolved oxygen levels are low, would be expected to become oxidized to nitrate-N as it travels further from the wastewater source; so this finding supports the use of nitrate-N as a historical wastewater indicator. Caffeine concentrations were more strongly correlated with EDCs. However, because caffeine has not been routinely monitored, nitrate-N remains the best historical proxy for EDCs in a cancer study.

The major advantages of our study are that extensive interview data allow for control of confounding by established and hypothesized breast cancer risk factors and that our exposure assessment method improves on previous breast cancer and drinking water studies, many of which are ecologic or include a single exposure measure, often after diagnosis. Study results are unlikely to be affected by bias in ascertainment of cases; because cases were identified from MCR, which has nearly complete reporting [40]. Exposure assessments are unlikely to be affected by self-report bias or observation bias; address histories and personal water behavior are the only aspects of the exposure measure that rely on self-report. Exposure scores were assigned using a geographic information system independent of any knowledge of disease status.

The study also contributes to a sparse literature. Although drinking water may contain carcinogens and EDCs and is the subject of numerous cancer studies, we identified only 11 epidemiologic studies in a Medline query for studies of drinking water and breast cancer. Overall, the studies suffer from exposure assessment in broad categories based on drinking water supply at diagnosis or death and do not adequately control for confounding. A meta-analysis of four individual-level case-control studies of chlorinated drinking water reported a relative risk of 1.18 (95% CI 0.90, 1.54) associated with chlorination; the power to detect a relative risk of 1.20 at *p *less than 0.05 was 0.27 [41]. A mutagenic constituent of disinfection byproducts, 3-chloro-4-(dichloromethyl)-5-hydroxy-2(5H)-furanone (known as mutagen -X or MX), has been identified as a mammary carcinogen [42]. The PCE Study [29,43] on Cape Cod observed higher risk in women who were accidentally exposed to perchloroethylene leaching from improperly prepared drinking water distribution pipes (relative exposure > 75th percentile compared to none: Adjusted OR = 1.6 (95% CI 1.1–2.4); > 90th percentile: Adjusted OR = 1.3 (95% CI 0.7–2.6)). A study of Wisconsin women did not find an association between adult exposure to drinking water contaminated by atrazine and breast cancer[44]

Although we were interested in nitrate-N as a proxy for EDCs from wastewater, nitrate-N itself has been hypothesized to increase risk for multiple cancers and birth outcomes. Elevated risk has been reported for colon cancer and infant neural tube defects at nitrate-N exposure levels below the US regulatory limit for drinking water; but, overall, health effects of nitrate-N in drinking water are poorly understood [45]. Thus, the exposure assessment developed in this study could be applied in studies of other health outcomes.

More that thirty years after the passage of the Safe Drinking Water Act in 1974 required suppliers to begin routine testing, these measurements remain a largely underutilized resource for individual-level studies of the health effects of environmental pollutants. Development of more advanced GIS-based exposure assessments linking routinely measured drinking water parameters with epidemiologic interview data could enhance studies of multiple health outcomes.

## Conclusion

We did not find evidence of an association between breast cancer incidence and drinking water contaminated by wastewater in a case-control study of Cape Cod, Massachusetts, women. Few other breast cancer studies have investigated exposures from drinking water, which may be contaminated by chemicals identified as mammary carcinogens or endocrine disruptors. Measurements required by the Safe Drinking Water Act are an under-used resource for investigating breast cancer and other long-latency health outcomes by integrating historical environmental data with interviews.

## Abbreviations

CI, confidence interval

CMS, Centers for Medicare and Medicaid Services

DES, diethylstilbestrol

EDC, Endocrine disrupting compound

EPA, Environmental Protection Agency

GIS, geographic information systems

MA DEP, Massachusetts Department of Environmental Protection

MCR, Massachusetts Cancer Registry

MX, 4-(dichloromethyl)-5-hydroxy-2(5H)-furanone

Nitrate-N, nitrate nitrogen

OR, odds ratio

PAH, polycyclic aromatic hydrocarbon

PCE, perchloroethylene, tetrachloroethylene

USGS, US Geological Survey

ZOC, zone of contribution

## Competing interests

The author(s) declare that they have no competing interests.

## Authors' contributions

JGB directed the study and drafted the manuscript. AA provided a portion of the data and advised the epidemiologic analysis. WMcK conducted the epidemiologic analysis. CHS designed the drinking water exposure assessment. TK conducted the GIS analysis. RAR advised in the design of the exposure assessment and interpretation of results. The authors contributed equally to this work. All authors participated in editing, and read and approved the final manuscript.
